# Immunogenicity of a third dose of the BNT162b2 COVID-19 vaccine in patients with CLL: effects on treatment selection

**DOI:** 10.1007/s00277-022-05003-6

**Published:** 2022-10-22

**Authors:** Panagiotis T. Diamantopoulos, Christina-Nefeli Kontandreopoulou, Christos Stafylidis, Dimitra Vlachopoulou, Nefeli Giannakopoulou, Maria Vardaka, Anthi Mpouhla, Eleni Variami, Athanasios Galanopoulos, Vassiliki Pappa, Mina Psichogiou, Angelos Hatzakis, Nora-Athina Viniou

**Affiliations:** 1 Hematology Unit, First Department of Internal Medicine, Laikon General Hospital, National and Kapodistrian University of Athens, Athens, Greece; 2 Department of Clinical Hematology, ‘G. Gennimatas’ District General Hospital, Athens, Greece; 3 Haematology Division, Second Department of Internal Medicine, Attikon General Hospital, National and Kapodistrian University of Athens, Athens, Greece; 4 Department of Hygiene, Epidemiology and Medical Statistics, Medical School, National and Kapodistrian University of Athens, Athens, Greece

**Keywords:** Chronic lymphocytic leukemia, BNT162b2 mRNA COVID-19 vaccine, Booster/3rd dose, Venetoclax, Ibrutinib

## Abstract

Patients with chronic lymphocytic leukemia (CLL) show suboptimal responses to the vaccines against SARS-CoV-2; it has been shown though that a booster dose of the BNT162b2 vaccine may lead to a significant increase in the seroconversion rates of immunocompromised patients. We conducted a prospective, non-interventional study to evaluate the immunogenicity of a third dose of the BNT162b2 vaccine in adult patients with CLL. Sera were tested before the first, after the second, and before and after the third dose for anti-SARS-CoV-2 receptor binding domain (RBD) spike protein IgG (anti-RBD). Thirty-nine patients with CLL were included in the study. The seroconversion rate increased from 28.2% before the third dose to 64.1% after the third dose and was higher in treatment-naïve patients (72.7% versus 47.1% in actively treated patients, *p* = 0.042). All but one patient achieving a seroconversion after the second dose retained after the third, while eight patients not achieving a seroconversion after the second dose (38.1%), did so after the third. Moreover, patients actively treated with venetoclax had a higher seroconversion rate than those treated with ibrutinib (87.5% versus 14.3%, *p* = 0.001). This study confirms the beneficial effect of a third dose of the BNT162b2 vaccine on the seroconversion rate in patients with CLL. Our results also strongly suggest that the use of venetoclax is correlated with higher immunogenicity/seroconversion rates than that of ibrutinib, a finding that has been reported by another study. A treatment strategy change during the pandemic favoring the use of venetoclax may be suggested based on our results, although these results should be validated in larger studies.

## Introduction

Patients with chronic lymphocytic leukemia (CLL) are vulnerable to severe infection for the severe acute respiratory syndrome coronavirus 2 (SARS-CoV-2), with high mortality rates [1–5]. Given the immune deregulation inherent to CLL but also aggravated by the treatment, response of patients with CLL to COVID-19 vaccines has been shown to be suboptimal [6–11]. Hence, strategies optimizing response to SARS-CoV-2 vaccination is crucial in these patients. Among the available strategies, a booster dose of the vaccine has been proven effective in immunocompetent individuals since trials have shown that a third dose of the BNT162b2 COVID-19 mRNA vaccine increases the antibody levels against the spike protein of SARS-CoV-2 [12–15].

Additionally, in studies of solid-organ (mainly kidney) transplant recipients, following a third mRNA vaccine dose, seroconversion has been observed in rates ranging from 30 to 49% of previously seronegative patients that received the standard two-dose regimen [16–19], while similarly designed studies have provided conflicting results in patients with CLL [20, 21].

In an in-press study from our group, we showed that patients with CLL have suboptimal response to the BNT162b2 vaccine [11]. In the present study, we assessed the immunogenicity of a third dose of the BNT162b2 COVID-19 vaccine in patients with CLL.

## Methods

### Patients

This is an extension of a previous study on the immunogenicity and safety of two doses of the BNT162b2 mRNA COVID-19 vaccine in patients with CLL [11]. Adult patients with CLL from three tertiary hospitals in Athens, Greece, vaccinated against SARS-CoV-2 with three doses of the BNT162b2 mRNA COVID-19 vaccine participated in the present study after providing a written informed consent. All patients had participated in the first phase of the study with the first two doses of the vaccine. Exclusion criteria included known human immunodeficiency virus infection, vaccination with other anti-SARS-CoV2 vaccines, and inability to provide written informed consent. All patients were approached by the treating physicians and enrolled in a consecutive manner. The study started on October 12, 2021, and its duration was 6 months. At baseline, the epidemiological, clinical, and laboratory characteristics of the patients as well as treatment data were recorded as follows. Age and disease stage at the time of vaccination, disease duration, complete blood count parameters (hemoglobin level, lymphocyte, neutrophil, monocyte, and platelet count), and gamma-globulin levels were recorded and analyzed. Moreover, data on the treatment of the patients (treatment lines, previous treatment with anti-CD20 antibodies, fludarabine, ibrutinib, or venetoclax, active treatment, and treatment regimen at the time of vaccination) were also collected and analyzed.

### Vaccination

Patients were vaccinated with the third 30-mcg dose of the BNT162b2 mRNA COVID-19 vaccine administered intramuscularly in the deltoid muscle, according to the national program for vaccination against SARS-CoV-2. The time interval between the second and the third dose of the vaccine was also recorded.

### Study procedures

This is a prospective non-interventional study designed to assess immunogenicity against SARS-CoV-2 at baseline (i.e., within 5 days before the third dose of the vaccine) and within 12–21 days after the third dose of the vaccine. Blood samples were collected at the predefined time points and sera were obtained after centrifugation and stored at − 80 °C.

Sera were tested for anti-SARS-CoV-2 receptor-binding domain (RBD) spike protein IgG (anti-RBD), using the Abbott SARS-CoV-2 IgG II Quant assay (Abbott Laboratories, Abbott Park, IL, USA), a two-step chemiluminescent microparticle immunoassay for the qualitative/quantitative detection of IgG antibodies against the RBD of the S1 subunit of the spike protein in human serum and plasma on the Architect i system. The details of the method and its clinical sensitivity and specificity have been provided elsewhere [11, 22, 23]. The assay threshold of 50 AU/mL was set as the seroconversion cutoff in the present study.

The study was approved by the Institutional Review Boards of all three participating centers (Laikon General Hospital, Athens, Greece, 01/22/2021, Attikon Hospital, Athens, Greece 02/24/2021, G. Gennimatas General Hospital, Athens, Greece, 02/18/2021).

### Statistical analysis

The IBM SPSS statistics, version 26 (IBM Corporation, North Castle, NY, USA), was used for the statistical analysis of the results. The Pearson chi-square test was used to test for associations between categorical variables, and the Fisher’s exact test was used instead for associations with less than five values in each category. The independent-samples Mann–Whitney *U* test was used for testing between a categorical variable with two levels and not normally distributed continuous variables, and the Kruskal–Wallis *H* test for categorical variables with more than two levels. The level of significance for all statistical tests was set at a probability value lower than 5% (2-sided *p* < 0.05).

## Results

Among 61 patients with CLL participating in the first study, thirty-nine patients vaccinated with the third dose of the BNT162b2 mRNA COVID-19 vaccine were included in the present analysis. The remaining 22 patients either refused to get vaccinated with the third dose (*N* = 5) or were vaccinated with another vaccine (*N* = 11) or were not vaccinated with the third dose during the predefined 6-month duration of the study (*N* = 6). All patients had measurements of the antibody titers before the first and after the second dose of the vaccine, as well as before and after the third dose of the vaccine. The main epidemiologic, clinical, laboratory, and treatment characteristics of the patients are shown in Table 1.

**Table 1:** Patient characteristics

Characteristic	Result
Number of patients, *N* (%)	39 (100)
Gender (male/female), *N* (%)	17/22 (43.6/56.4)
Age (years), median (range)	73.0 (41.0–88.0)
RAI stage, *N* (%)	
0	7
1	8
2	9
3	11
4	4
Previous treatment, *N* (%)	23 (59.0)
Previous treatment lines, *N* (%)	
0	16 (41.1)
1	8 (20.5)
2	6 (15.4)
3	3 (7.7)
≥ 4	6 (15.4)
Previous treatment lines, *N* (%)	
≤ 2	30 (76.9)
> 2	9 (23.1)
Number of previous treatments, median (range)	1 (0–5)
Previous anti-CD20, *N* (%)	11 (28.2)
Time from last dose of anti-CD20 to:	
First vaccine dose	33.1 (6.7–89.9)
Third vaccine dose	38.5 (12.0–95.0)
Previous fludarabine, *N* (%)	1 (2.6)
Previous ibrutinib, *N* (%)	2 (5.1)
Previous venetoclax, *N* (%)	0 (0.0)
Actively treated, N (%)	17 (43.6)
Type of treatment, *N* (%)	
Chlorambucil	2 (11.8)
Ibrutinib	7 (41.2)
Venetoclax	8 (47.1)
Hemoglobin level (g/dL), median (range)	13.4 (9.1–16.3)
Lymphocyte count (× 10^9^/L), median (range)	8.62 (0.57–240.89)
Platelet count, (× 10^9^/L), median (range)	163 (36–327)
Gamma globulin level (g/dL), median (range)	7.4 (1.6–29.28)

Pre-3rd dose samples were obtained within a median time of 2 (range, 0–5) days before the third dose of the vaccine, while post-3rd dose samples were obtained within a median time of 14 (range, 12–19) days after the third dose of the vaccine.

The median time between the second and third dose of the vaccine was 5.6 (range, 2.0–7.7) months.

### Immunogenicity/seroconversion results

The median post-2nd dose antibody titer was 28.6 AU/mL (0.0–40,000) with 18 patients (46.2%) achieving seroconversion, while the median pre-3rd dose antibody titer was 10.5 AU/mL (0.0–7869.2) with the seroconversion rate declining to 28.2% (11 patients). Out of 18 patients who achieved seroconversion with the first two doses of the vaccine, 10 (55.5%) retained this status before the 3rd dose, while out of 21 patients not seroconverted after the first two doses, seroconversion was detected in only one before the third dose, implying a natural infection during the time interval between the two samples collections, although there was no report of COVID19 in any of the patients.

**Fig. 1:**
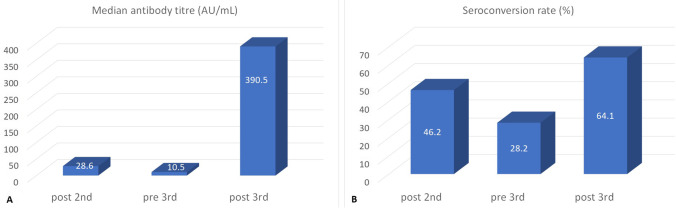
Immunogenicity (**A**) and seroconversion rate (**B**) after the second, before and after the third dose of the BNT162b2 vaccine against SARS-CoV-2 in 39 patients with chronic lymphocytic leukemia

The median post-3rd dose antibody titer was 390.5 AU/mL (1.1–39,264.7) with 25 patients (64.1%) achieving seroconversion (Fig. 1, Table 2). The seroconversion rate was not correlated with any of the studied baseline characteristics of the patients (age, gender, RAI stage, hemoglobin and gamma-globulin level, lymphocyte and platelet count), while treatment-naïve patients had a higher seroconversion rate (16/22, 72.7%) than actively treated patients (8/17, 47.1%), *p* = 0.042.

**Table 2:** Immunogenicity and seroconversion results

Characteristic	Pre-1st dose	Post-2nd dose	Pre-3rd dose	Post-3rd dose
Antibody titer (AU/mL), median (range)	0.0 (0.0–557.5)	39.3 (0.0–40,000)	22.8 (0.0–7.869.2)	1734.1 (1.1–39,263.6)
Seroconversion rate, *N* (%)	1 (2.6)	18 (46.2)	11 (28.2)	25 (64.1)
Seroconversion rate in ibrutinib treated patients, *N* (%)	0/5 (0.0)	1/5 (20.0)	0/7 (0.0)	1/7 (14.3)
Seroconversion rate in venetoclax treated patients, *N* (%)	0/8 (0.0)	5/8 (62.5)	3/8 (37.5)	7/8 (87.5)

Among 21 patients not achieving seroconversion with the first two doses of the vaccine, 8 (38.1%) achieved seroconversion after the third dose, while only one patient, who achieved seroconversion with the first two doses, lost it after the third. This was a 65-year-old treatment-naïve female with worsening hypogammaglobulinemia.

In patients who achieved seroconversion after the 2nd dose of the vaccine and retained it after the 3rd dose (*N* = 17), there was a non-statistically significant increase in the antibody titer (10,593.6 AU/mL versus 6981.0 AU/mL, *p* = 0.109). Since the study was not designed to record COVID-19, but only immunogenicity results, there was no active monitoring for COVID-19 in the study population. The median follow-up of the patients since the first sample collection was 7.3 months (range 3.8–11.1).

**Fig. 2:**
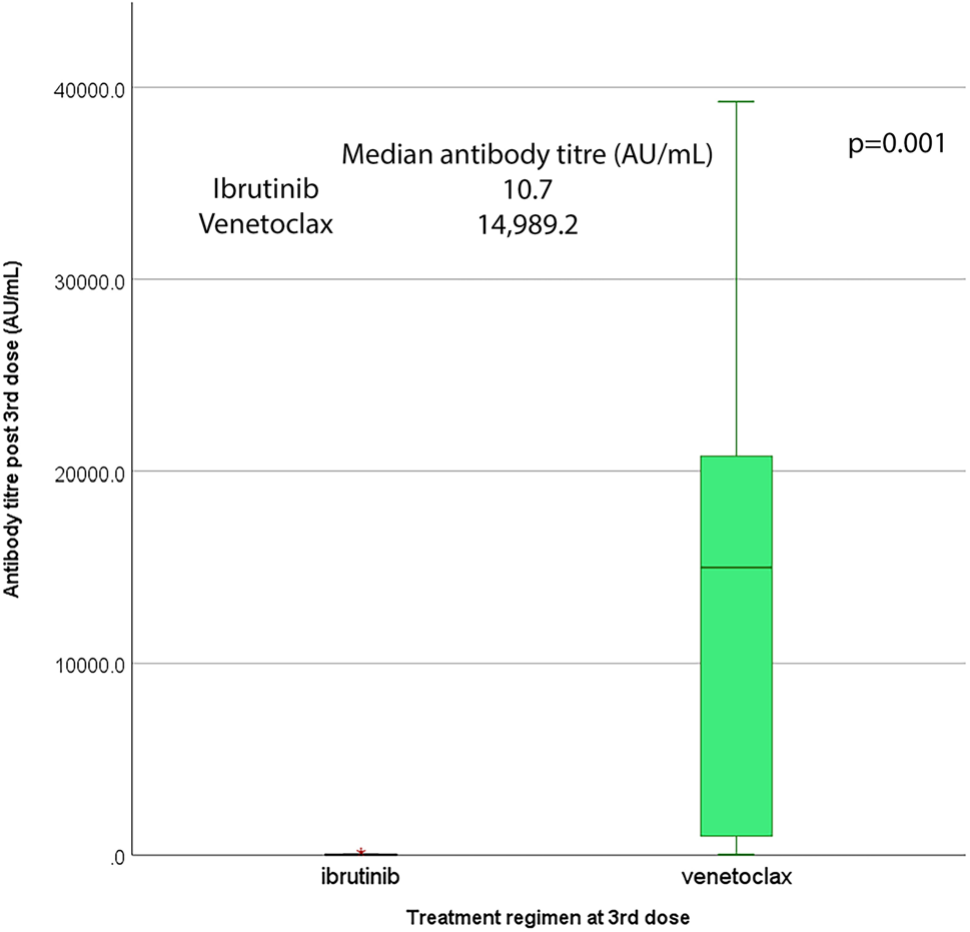
Titres of the antibody against the spike protein of SARS-CoV-2 in patients with chronic lymphocytic leukemia treated with ibrutinib and venetoclax monotherapy after the third dose of the BNT162b2 vaccine against SARS-CoV-2

Among 17 actively treated patients, seroconversion was achieved in 8 (44.4%). Fifteen out of 17 actively treated patients were treated with either ibrutinib (*N* = 7; median duration of treatment, 16.5 months; range 5–45 months) or venetoclax monotherapy (*N* = 8; median duration of treatment 29, months; range 15–29 months). Among those patients, there was a statistically significant difference of seroconversion after the 3rd dose (1/7 ibrutinib treated patients versus 7/8 venetoclax treated patients achieved seroconversion, *p* = 0.005), with a corresponding statistically significant difference in the antibody titers between the two groups (10.7 AU/mL versus 14,989.2 AU/mL respectively, *p* = 0.001, Fig. 2, Table 2). Ibrutinib- and venetoclax-treated patients did not differ in terms of RAI stage (*p* = 0.813), hemoglobin level (*p* = 0.980), lymphocyte (*p* = 0.132) or platelet count (*p* = 0.315), gamma globulin levels (*p* = 0.315), or treatment duration (*p* = 0.592).

## Discussion

Administering a booster dose of a vaccine is a common practice, after initial immunization [24–27], to provide a re-exposure to the immunizing antigen and consequently increase immunity against the antigen. The need for a booster dose of the vaccines against SARS-CoV-2 was evidenced by studies on the kinetics of the antibodies against the spike protein of the virus, showing that they declined after several months from the first exposure to the antigen [28–31]. Thus, a booster dose for all available vaccines against SARS-CoV-2 became a priority for immunocompetent and immunocompromised hosts. The well-established results of the booster dose of the BNT162b2 mRNA COIVD-19 vaccine in the general population [32–34] have been challenged in patients with immunosuppression, such as malignancies, transplantation, or hematologic diseases, and several studies have tried to assess the immunogenicity and efficacy of the third dose of the vaccine in these populations.

In September 2021, encouraging results from a study in 160 kidney transplant recipients and 20 patients with CLL vaccinated with a third dose of an mRNA COVID-19 vaccine showed that the antibody levels were moderately increased; nevertheless, this increase might not be clinically significant since few patients reached a threshold associated with vaccine effectiveness [35]. Another study showed that 23.8% of 172 patients with CLL who failed to respond to the first two doses of the BNT162b2 mRNA COIVD-19 vaccine responded to a third dose [20]. On the contrary, in another study in patients with several hematologic malignancies, none of the 15 evaluated patients with CLL responded to the third dose of the vaccine [21].

In our study, 38.1% of patients not achieving seroconversion after the second dose of the BNT162b2 mRNA COVID-19 vaccine achieved seroconversion after the third dose. Moreover, all but one patient (94.4%), who achieved seroconversion after the second dose, retained it after the booster dose, with a non-statistically significant trend for higher antibody titers. These results along with previously published studies justify the administration of a booster dose in patients with CLL and probably encourage the administration of a fourth dose in these patients.

In accordance with previous studies, treatment-naïve patients had higher seroconversion rates than actively treated patients [20, 36, 37]. A clinically significant result in our study was the noticeable difference in the seroconversion rate among patients under treatment with ibrutinib and venetoclax, favoring the latter. This result was also highlighted in the results of the first phase of the study after the first two doses of the vaccine, but in the present study is even more pronounced and in accordance with the results of a recent study that showed that patients under venetoclax monotherapy achieved significantly higher response rates than those treated with Bruton tyrosine kinase inhibitor (BTKi) or BTKi in combination with anti-CD20 monoclonal antibodies or venetoclax [38]. This finding, until otherwise proven, may justify a temporary shift in the treatment paradigm for patients with CLL, favoring the administration of venetoclax over BTKi, during the pandemic, given that the data is really strong, but also taking into consideration the small number of patients included in the analysis.

The strengths of the present study are the inclusion of a homogeneous population of patients with CLL vaccinated with only one type of anti-SARS-CoV-2 vaccine, as well as the analysis of patients treated with ibrutinib and venetoclax monotherapy that yielded a decision-making result that may affect treatment selection in patients with CLL. The main limitation of this study is the small patient sample that did not permit further statistical analyses. The small patient sample may also explain the lack of correlation of seroconversion rate with the baseline characteristics of the patients.

Finally, an inherent limitation of the study is the lack of detection of cellular immune responses in the studied population. It has been reported by several authors that the impaired humoral response in patients with CLL vaccinated against SARS-CoV-2 may be counteracted by an effective cellular response. Thus, it has been shown that even though serological response is severely impaired in patients with CLL especially when they are treated with rituximab or ibrutinib, a significant proportion of the patients may develop sufficient cell-mediated immunity [39]. Moreover, although early cellular immune responses rely mostly on CD8 + T cells and these cells are significantly increased after vaccination, they still remain lower than normal, and this may play a role in the reduced memory response and the need for booster doses, especially in immunocompromised hosts such as patients with CLL [40]. On the other hand, there are reports of unaffected numbers of specific CD8 + T cells against SARS-CoV-2 after vaccination in patients with CLL [41].

In conclusion, patients with CLL may benefit from a booster dose of the BNT162b2 mRNA COVID-19 vaccine, since more than a third of patients not achieving seroconversion with the first two doses, seroconverted after the third. Moreover, venetoclax monotherapy seems to be a valuable treatment choice for patients with CLL during the pandemic for yet another reason, apart from its efficacy and favorable safety profile; the high rates of seroconversion after vaccination against SARS-CoV-2.

## Data Availability

Data on the experiments and patient data are available upon reasonable request. For original data, please contact pandiamantopoulos@gmail.com.
